# Supplementary material for: End Sequence Analysis Toolkit (ESAT) expands the extractable information from single-cell RNA-seq data
Source: Genome Res. 2016 Oct;26(10):1397–410. doi: 10.1101/gr.207902.116 (PMC5052061; doi:10.1101/gr.207902.116)
Supplement: Supplemental Material [file supp_gr.207902.116_Supp_Materials.tar.gz › ESAT-esat_v0.1/doc/html/classbroad_1_1core_1_1siphy_1_1_evolutionary_model-members.html]

ESAT: Member List


ESAT


- Main Page
- Related Pages
- Packages
- Classes
- Files

- Class List
- Class Index
- Class Hierarchy
- Class Members

All Classes Namespaces Files Functions Variables Enumerator Pages

- broad
- core
- siphy
- EvolutionaryModel

broad.core.siphy.EvolutionaryModel Member List

This is the complete list of members for broad.core.siphy.EvolutionaryModel, including all inherited members.

|  |  |  |
| --- | --- | --- |
| changeBackground(double pA, double pC, double pG, double pT) | broad.core.siphy.EvolutionaryModel |  |
| changeBackground(Matrix newPi) | broad.core.siphy.EvolutionaryModel |  |
| computeJMatrix(PhylogenyNode node) | broad.core.siphy.EvolutionaryModel |  |
| computeLikelihood(Map< String, Matrix > leafValues, PhylogenyNode root, int site) | broad.core.siphy.EvolutionaryModel |  |
| computeTransitions(double branchLength) | broad.core.siphy.EvolutionaryModel |  |
| copy() | broad.core.siphy.EvolutionaryModel |  |
| EvolutionaryModel(EvolutionaryModelParameters parameters) | broad.core.siphy.EvolutionaryModel |  |
| evolve(String sequence) | broad.core.siphy.EvolutionaryModel |  |
| fitOmega(Map< String, Matrix > leafValues, Phylogeny tree, int window) | broad.core.siphy.EvolutionaryModel |  |
| fitPI(Map< String, Matrix > alignmentColumn, Phylogeny tree) | broad.core.siphy.EvolutionaryModel |  |
| getAlphabetSize() | broad.core.siphy.EvolutionaryModel |  |
| getModelParameters() | broad.core.siphy.EvolutionaryModel |  |
| getNodeLikelihoodParameterMap() | broad.core.siphy.EvolutionaryModel |  |
| getParameters() | broad.core.siphy.EvolutionaryModel |  |
| getPi() | broad.core.siphy.EvolutionaryModel |  |
| getPVal(OmegaFit fit, boolean useLogOddsLikelihood) | broad.core.siphy.EvolutionaryModel |  |
| getRateMatrix() | broad.core.siphy.EvolutionaryModel | protected |
| getTree() | broad.core.siphy.EvolutionaryModel |  |
| omegaEMIteration(Map< String, Matrix > leafValues, PhylogenyNode root, double newOmega, int window) | broad.core.siphy.EvolutionaryModel |  |
| piEMIteration(Map< String, Matrix > column, PhylogenyNode root, Matrix newPI) | broad.core.siphy.EvolutionaryModel |  |
| pruneAndPeel(Map< String, Matrix > leafValues, PhylogenyNode root, int site) | broad.core.siphy.EvolutionaryModel |  |
| pruneTree(List< String > ignoreList) | broad.core.siphy.EvolutionaryModel |  |
| resetPI() | broad.core.siphy.EvolutionaryModel |  |
| RPIDecomposition() | broad.core.siphy.EvolutionaryModel |  |
| sample() | broad.core.siphy.EvolutionaryModel |  |
| sample(PhylogenyNode node, short parentState) | broad.core.siphy.EvolutionaryModel |  |
| setAlphabetSize(int alphabetSize) | broad.core.siphy.EvolutionaryModel |  |
| setModelParameters(EvolutionaryModelParameters parameters) | broad.core.siphy.EvolutionaryModel |  |
| setNeutralOmegaDistribution(String neutralOmegaFile) | broad.core.siphy.EvolutionaryModel |  |
| setOmega(double omega) | broad.core.siphy.EvolutionaryModel |  |
| setOmegaDistByTreeLength(String neutralOmegaDistributionByTreeLengthFile) | broad.core.siphy.EvolutionaryModel |  |
| setParameters(EvolutionaryModelParameters parameters) | broad.core.siphy.EvolutionaryModel |  |
| setPi(Matrix pi) | broad.core.siphy.EvolutionaryModel |  |


---

Generated on Fri Aug 15 2014 15:10:57 for ESAT by  

 1.8.7
